# NYESO-1/LAGE-1s and PRAME Are Targets for Antigen Specific T Cells in Chondrosarcoma following Treatment with 5-Aza-2-Deoxycitabine

**DOI:** 10.1371/journal.pone.0032165

**Published:** 2012-02-27

**Authors:** Seth M. Pollack, Yonqing Li, Megan J. Blaisdell, Erik A. Farrar, Jeffrey Chou, Benjamin L. Hoch, Elizabeth T. Loggers, Eve Rodler, Janet F. Eary, Ernest U. Conrad, Robin L. Jones, Cassian Yee

**Affiliations:** 1 Clinical Research Division, Fred Hutchinson Cancer Research Center, Seattle, Washington, United States of America; 2 Department of Oncology, University of Washington, Seattle, Washington, United States of America; 3 Department of Pathology, University of Washington, Seattle, Washington, United States of America; 4 Group Health Research Institute, Seattle, Washington, United States of America; 5 Department of Radiology, University of Washington, Seattle, Washington, United States of America; 6 Department of Orthopedics and Sports Medicine, University of Washington, Seattle, Washington, United States of America; Saint Louis University School of Medicine, United States of America

## Abstract

**Background:**

Chondrosarcoma has no proven systemic option in the metastatic setting. The development of a non-cross-resistant strategy, such as cellular immunotherapy using antigen-specific T cells would be highly desirable. NY-ESO-1 and PRAME are members of the Cancer Testis Antigen (CTA) family that have been identified as promising targets for T cell therapy. LAGE-1 is a cancer testis antigen 90% homologous to NY-ESO-1, sharing the 157–165 A*0201 NY-ESO-1 epitope with its transcript variant, LAGE-1s. A number of CTA's have been induced using 5-Aza-2-Deoxycitabine (5-Aza-dC) in other cancers. We sought to evaluate the feasibility of targeting chondrosarcoma tumors using NY-ESO-1/LAGE-1s and PRAME specific T cells using 5-Aza-dC to induce antigen expression.

**Methods:**

We used 11 flash frozen tumors from the University of Washington tumor bank to test for the expression of NY-ESO-1, PRAME, LAGE-1s and LAGE-1L in chondrosarcoma tumors. Using four chondrosarcoma cell lines we tested the expression of these CTA's with and without 5-Aza-dC treatments. Finally, using NY-ESO-1/LAGE-1s and PRAME specific effectors that we generated from sarcoma patients, we evaluated the ability of these T cells to lyse A*0201 expressing chondrosarcoma cell lines in vitro both with and without 5-Aza-dC treatment.

**Results:**

A minority (36%) of chondrosarcoma tumors expressed either NY-ESO-1 or LAGE-1s at >10% of our reference value and none expressed PRAME at that level. However, in all four of the chondrosarcoma cell lines tested, NY-ESO-1 and PRAME expression could be induced following treatment with 5-Aza-dC including in cell lines where expression was absent or barely detectable. Furthermore, NY-ESO-1/LAGE-1s and PRAME specific CD8+ effector T cells were able to specifically recognize and lyse A*0201 expressing chondrosarcoma cell lines following 5-Aza-dC treatment.

**Conclusion:**

These data suggest that adoptive immunotherapy in combination with 5-Aza-dC may be a potential strategy to treat unresectable or metastatic chondrosarcoma patients where no proven systemic therapies exist.

## Introduction

Chondrosarcoma comprises approximately 20–30% of primary malignant bone neoplasms with an annual incidence close to 1 in 200,000 person/years. [1]. In the setting of localized disease, surgery is usually curative [2]. However, in the metastatic setting or in the case of unresectable disease, prognosis is poor and there are no proven systemic options [3] [4], [5]. The development of a non-cross-resistant strategy, such as cellular immunotherapy using antigen-specific T cells would be highly desirable. Antigen specific adoptive T cell therapy involves the ex vivo selection and expansion of tumor antigen-specific T cells which can then be re-infused to treat patients and represents an increasingly effective modality for the treatment of patients with refractory cancers. Phase I and II trials of adoptive T cell therapy for metastatic melanoma have yielded impressive results [6], [7], [8].

With the exception of synovial sarcoma, the identification of immunogenic target antigens for sarcomas has not been widely explored. The family of Cancer Testis Antigens (CTA's) has been cited as potential target antigens in sarcomas. These immunogenic antigens are frequently expressed by the developing embryo but are not expressed in significant quantities in the normal adult other than in the testis and placenta [9]. Solid tumor malignancies such as melanoma have been successfully treated by targeting CTA's in early phase immunotherapy trials [8], [10]. The NCI Surgery Branch recently published their experience treating 6 synovial sarcoma patients using T-cells with a transduced T-cell receptor targeting NY-ESO-1 [11].

The CTA LAGE-1, which is 90% homologous to NY-ESO-1, has two transcript variants LAGE-1s and LAGE-1L. The transcript variant LAGE-1s shares the A*0201 epitope, SLLMWITQC , with NY-ESO-1 and is thus an equivalent target for NY-ESO-1 directed therapy. Here, we explore the possibility of targeting chondrosarcoma using the SLLMQITQC epitope of NY-ESO-1 (157–165) and LAGE-1s . The expression of another cancer testis antigen, PRAME, [12], [13] and its associated A*0201 epitope ALYVDSLFFL ((300–309) was also evaluated.

Expression of CTA expression can be subject to epigenetic control and up-regulated using hypo-methylating agents. Of note, MAGE-A3 induction can be unregulated in lung cancer and esophageal cancer cell lines using 5-Aza-2-Deoxycitabine (5-Aza-dC) [14], [15] and has been shown to facilitate recognition by NY-ESO-1 and PRAME specific CTL in several other malignancies [16], [17], [18], [19]. In a phase I clinical trial of 5-Aza-dC for the treatment of patients with lung and esophageal cancers, expression of NY-ESO-1 and MAGE-was up-regulated following treatment and lasted at least 8 months following therapy [20].

We evaluated if the immunogenic CTA's, NY-ESO-1, LAGE-1s and/or PRAME represent feasible targets in chondrosarcoma for antigen-specific T cell therapy.

## Materials and Methods

### Tumor Samples and Ethics Statement

All blood and tumor samples used in this study were obtained under either the Fred Hutchinson Cancer Research Center or the University of Washington Institutional Review Board approved protocols. Written consent was obtained from all patients who contributed samples.

Human chondrosarcoma samples were obtained through the UW sarcoma tumor bank (IRB approved protocol #21369). Patients with extra skeletal myxoid chondrosarcoma or synovial chondromatosis were excluded from analysis. Although some patients had multiple banked samples, only one sample per patient was included.

### Cell Lines and treatment with 5-Aza-2-Deoxycitabine (5-Aza-dC)

The chondrosarcoma cell lines JJ, FS and 105KC were gifts of Dr. Joel Block (Rush University) [21], [22]. Cells were maintained in 40% Dulbecco's modified Eagle's medium (high glucose), 40% Minimal Essential Media, 10% F12 (Gibco), 10% fetal bovine serum plus (Gemini Bio-Products Woodland, CA) 100 nM hydrocortisone (Sigma, St. Louis, MO), 100 ng/mL insulin (Gibco) and 25 mg/mL of ascorbic acid and either 0.05 mg/mL of gentamycin (Gibco) or 50 U/ml penicillin, 50 mg/ml streptomycin (Invitrogen Life Technologies, Carlsbad, CA). The cell line SW1353 was purchased from American Type Culture Collection (Manassas, MD) and was maintained in 90% Dulbecco's modified Eagle's medium (high glucose), 10% FBS and 50 U/ml penicillin, 50 mg/ml streptomycin (Invitrogen Life Technologies, Carlsbad, CA).

The melanoma cell lines Mel A375 (gift from S. Rosenberg) and Mel 526 (gift from M. Lotze) were used as reference samples and controls and were maintained in RPMI with Hepes (25 mM), L-glutamine (4 mM), penicillin (50 U/ml), streptomycin (50 mg/ml), sodium pyruvate (10 mM), nonessential amino acids (1 mM), and 10% fetal bovine serum (Hyclone, UT, USA). The TAP-deficient lymphocyte cell line T2 were maintained in RPMI 1640 containing 25 mM HEPES, 2 mM L-glutamine, 50 U/ml penicillin, 50 mg/ml streptomycin (Invitrogen Life Technologies, Carlsbad, CA), and 10% FBS.

For experiment using 5-Aza-dC, cells were treated with either 5-Aza-dC 1 µM (dissolved in PBS) or PBS vehicle (control). Media and either 5-Aza-dC or vehicle were changed every 12 hours for 48 hours and then allowed to rest for 48 hours before RNA extraction or functional analysis was performed. As a control, normal PBMC from an otherwise healthy sarcoma patient were also cultured with 5-Aza-dC and evaluated.

### HLA-Typing

High-resolution class I HLA-typing of tumor cell lines and sarcoma patients considered for leukapharesis was performed by the Puget Sound Blood Center.

### Generation of Antigen Specific Effectors

NY-ESO-1/LAGE-1s and PRAME specific effectors were generated in our lab from sarcoma patients who were HLA typed under IRB approved protocol (FCRC protocol #1765) and found to express A*0201. Patients underwent leukapheresis, also under IRB approved protocols (FHCRC protocol #1246).

PBMC derived dendritic cells [23] were pulsed with the NY-ESO-1/LAGE-1s peptide (the 157–165 epitope) SLLMWITQC or the PRAME peptide (the 300–309 epitope) ALYVDSLFFL. PBMC were depleted of CD25^+^ T cells using the CliniMACS CD25 MicroBeads (Miltenyi Biotech, Auburn, CA) according to manufacturer's instructions and were stimulated using IL-21 as previously described [24]. MHC tetramers made in our Immunologic Monitoring Core Facilities were used to sort tetramer (See Figure S1) positive populations which were then expanded using a Rapid Expansion Protocol [25] in the case of the PRAME specific cells or cloned and subsequently expanded in the case of the NY-ESO-1/LAGE-1s specific cells. MART-1 specific effectors were cloned by the same methods from a melanoma patient.

### Quantitative Real Time PCR

RNA was extracted from frozen tumor samples using Trizol (invitrogen) and from cell lines using RNeasy kit (Qiagen). Because banked tissue had been obtained under a variety of different conditions, RNA was checked prior to any analysis using either a gel or a bioanalyzer prior to ensure it had not degraded before further analysis was performed. RNA samples were converted to cDNA using the Transcriptor First Strand cDNA Synthesis Kit (Roche).

All primers used in these experiments are listed in Table 1. Initially as a screen to look for LAGE-1, a primer from SA Biosciences was used (the sequence is proprietary). Subsequently, we designed specific primers for the two transcript variants LAGE-1s and LAGE-1L. PRAME specific primers were designed using the website Primer Depot [26]. Results were analyzed using GAPDH as a house keeping gene and are calculated relative to either the cell line MelA375 (NY-ESO-1 and PRAME) or JJ (LAGE-1s and LAGE-1L) using the standard curve method. Amplification was performed using SYBR Green PCR Master Mix (Applied Biosystems, Foster City, Ca) on an ABI 7900HT (Applied Biosystems, Foster City, Ca).

**Table 1:** qRT-PCR primers used for these experiments.

Gene	Sequence	Reference
NY-ESO-1	5′-TGCTTGAGTTCTACCTGCCA-3′	[39]
	5′-TATGTTGCCGGACACAGTGAA-3′	
PRAME	5′-GCTTCAAAATGGAACGAAGG-3′	from primer depot:[26]
	5′-TGCCAGCTCCACAAGTCTC-3′	
LAGE-1s	5′-CGCCCATGGAAGCGGAGCTG-3′	designed for these studies
	5′-CTGCAGCAGTCAGTCGGATAAACA-3′	
LAGE-1L	5′-TGCTTCAGTTGCACATCACGATGC-3′	designed for these studies
	5′-TGGTCCCGAACTGACATAAACAGTAGG-3′	
GAPDH	5′-GAAGGTGAAGGTCGGAGTC-3′	[40]
	5′-GAAGATGGTGATGGGATTTC-3′	

For all qPCR experiments, the melanoma cell line Mel375 was used a reference for relative quantification of NY-ESO-1 and PRAME as this cell line strongly expresses both cell lines and can be lysed specifically by targeting each of these proteins. Similarly, JJ was selected as a reference for LAGE-1s as it does not express a significant quantity of NY-ESO-1 and expresses sufficient LAGE-1s such that it can by lysed using NY-ESO-1/LAGE-1s specific effectors.

### Chromium Release Assay and Cold Target Inhibition

Target cells were labeled with 100 µCi ^51^Cr and co cultured with effector cells for 4–6 hours at 37°C plus 5% CO_2_. To confirm that the effectors were lysing target tumor cells in an antigen specific fashion, cold target inhibition was performed [27]. Chromium labeled 5-Aza-dC treated tumor lines were incubated with non-radiolabelled T2 lymphocytes which were either unpulsed (control), pulsed the PRAME peptide, ALYVDSLFFL, or pulsed with the NY-ESO-1 peptide SLLMWITQC. Ratios of 30∶1 and 10∶1 unlabeled to ^51^Cr labeled targets were used [27]. E∶T ratios of 30∶1 were used. Conditions were otherwise similar to the other chromium release assays performed.

### Statistical Considerations

All *in vitro* experiments were repeated at a minimum of three times to be sure results were reproducible. Standard errors and p-values (calculated using student's T test) are calculated using multiple data points within a single representative experiments done in either triplicate or quadruplicate.

## Results

### A Minority of Chondrosarcoma Tumors Express Either LAGE-1s or NY-ESO-1

In order to characterize the expression pattern of NY-ESO-1, LAGE-1s and PRAME in chondrosarcoma tumors, we used qRT-PCR to test the tumors of eleven chondrosarcoma patients (Table 2).

**Table 2:** qRT-PCR results on flash frozen chondrosarcoma tumors from the UW Sarcoma Tissue Bank relative to positive Control Cell Lines Mel375 (for NY-ESO-1 and PRAME) and JJ (For LAGE-1s).

Patient	Histology	Patient Outcome	NY-ESO	PRAME	LAGE-1s
1	Conventional Chondrosarcoma Grade 2/3	metastatic disease	0.0%	0.1%	0.0%
2	Clear cell chondrosarcoma grade 1/3	metastatic disease	0.0%	0.2%	**21.7%**
3	Conventional chondrosarcoma grade 2/3	locally recurrent	0.0%	0.0%	0.3%
4	conventional chondrosarcoma grade 3/3	metastatic disease	1.5%	0.0%	**68.7%**
5	Conventional Chondrosarcoma low grade (1/3)	disease free	0.0%	0.2%	**17.4%**
6	Conventional Chondrosarcoma grade 2/3	disease free	**15.4%**	0.0%	0.0%
7	Dedifferentiated Chondrosarcoma (high grade)	metastatic disease	0.0%	3.4%	0.0%
8	Conventional Chondrosarcoma grade 3/3	metastatic disease	0.0%	0.0%	0.0%
9	Conventional Chondrosarcoma Grade 2/3	disease free	0.0%	0.0%	1.0%
10	Conventional Chondrosarcoma grade 2/3	local recurrence	0.0%	0.0%	0.0%
11	Conventional Chondrosarcoma grade 1/3	local recurrence	0.0%	0.0%	0.0%

Four of the 11 tumors had detectable expression of NY-ESO-1 however in all but one case, the level of expression was less than 2% of the reference sample Mel375 (in 2 of the cases it was less than 0.1% of the reference sample). There was one tumor that expressed NY-ESO-1 at over 15% of the reference samples. PRAME expression was similarly low, with detectable expression seen in 7 of the samples but at levels not more than 10% relative to Mel 375 in any of the samples.

There were three samples (27%) that had quantitative expression of LAGE-1s that was >10% with respect to the reference sample JJ. LAGE-1L was also examined (not shown) and the same 3 patients were the only patients with >10% expression of LAGE-1L compared to the reference sample. LAGE-1L does not share the SLLMWITQC epitope with NY-ESO-1 and LAGE-1s.

In summary, a total of four of eleven (36%) patients examined expressed either NY-ESO-1 or LAGE-1s at a quantitative level that might be suitable for targeting using antigen specific T cells targeting the HLA-A2-restricted NY-ESO-1/LAGE-1s epitope: SLLMWITQC.

### NY-ESO-1, PRAME and LAGE-1s Can be Up regulated in Chondrosarcoma Cell Lines Using 5-Aza-2-Deoxycitabine

To broaden the eligibility of potential chondrosarcoma patients for NY-ESO-1 and PRAME specific T-cell therapy, we evaluated whether 5-Aza-dC could be used to up-regulate these antigens in chondrosarcoma cell lines, FS, JJ, 105KC and SW1353.

**Figure 1:**
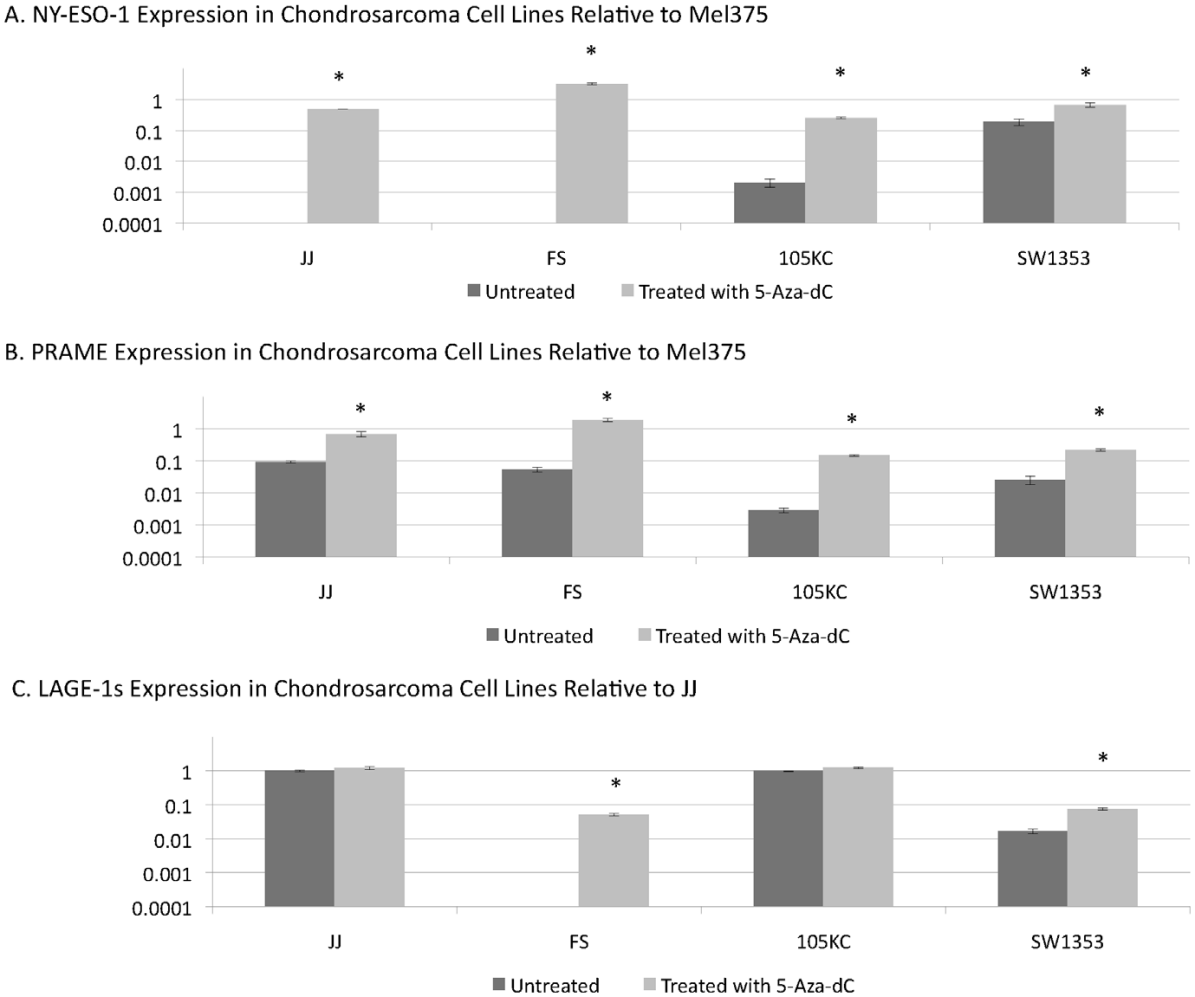
qRT-PCR expression in the chondrosarcoma cell line FS, JJ, 105KC and SW1353 with and without treatment using 5-Aza-dC for 48 hours. Treated cells rested 48 hours prior to RNA extraction. Primers are listed in Table 1. Data is presented relative to a reference sample: either Mel375 for NY-ESO-1 and PRAME or JJ for LAGE-1s normalized to GAPDH. Asterisk indicates statistical significance of p<0.05. Note that all CT antigens were significantly increased following 5-Aza-dC in all chondrosarcoma lines with the exception of LAGE-1s expression in the 105KC and JJ. These cell lines both strongly expresses LAGE-1s without treatment and were not changed significantly by 5-Aza-dC. The JJ cell line is not displayed in 1C as it is used for a reference value for LAGE-1s. Error bars describe variation between multiple values within a single experiment. Experiments were repeated at least three times to ensure reproducibility.

NY-ESO expression increased following 5-Aza-dC treatment in each of the four cell lines. Of note, in cell line FS, NY-ESO-1 was undetectable prior to treatment but increased to levels even higher than the reference sample following 5-Aza-dC treatment (Figure 1A). The cell line SW1353 expressed NY-ESO-1 at baseline when untreated and further increased expression following 5-Aza-dC treatment. The cell line 105KC had weak but detectable levels of NY-ESO-1 and the cell line JJ had barely detectable NY-ESO-1 expression (less than 0.0001 relative to Mel375). Both of these cell lines had multiple log fold increases in NY-ESO-1 expression following 5-Aza-dC treatment.

Similar increases were seen with regards to expression of PRAME (Figure 1B). Much like our clinical samples, in each cell line at baseline PRAME expressed in the untreated cell lines at a relatively low level. However PRAME expression was increased significantly following 5-Aza-dC treatment including the cell lines FS, which had increased expression above the level of the reference sample. The cell line JJ had expression of PRAME increase to near the level of the reference sample.

Both the cell line JJ and 105KC express significant amounts of LAGE-1s without treatment and expression was not significantly increased following 5-Aza-dC treatment. The cell line FS does not express LAGE-1s but expression was induced following 5-Aza-dC treatment. The cell line SW1353 had a low level of expression which increased following treatment (Figure 1C). Of note, LAGE-1L expression was also increased/induced in these cell lines including the cell line FS which had undetectable expression at baseline (data not shown).

Of note, normal PBMC from an otherwise healthy sarcoma patient were treated with 5-Aza-dC. Both NY-ESO-1 and PRAME were undetectable in both the treated and untreated samples. This is consistent with what has also been seen in normal fibroblast cell lines [28].

### 5-Aza-dC Increases Antigen Specific Recognition and Cell Lysis in Chondrosarcoma Cell Lines Treated with NY-ESO-1/LAGE-1s and PRAME Specific Effectors

T cell epitopes for several CTAs have been identified for HLA-A*0201, which is expressed in more than 40% of individuals. High resolution class I typing of all four chondrosarcoma cell lines and revealed that the FS and JJ cell lines both were positive for A*0201. The cell lines 105KC and SW1353 did not express A*0201 and were thus excluded from functional analysis using A*0201 positive effectors.

**Figure 2:**
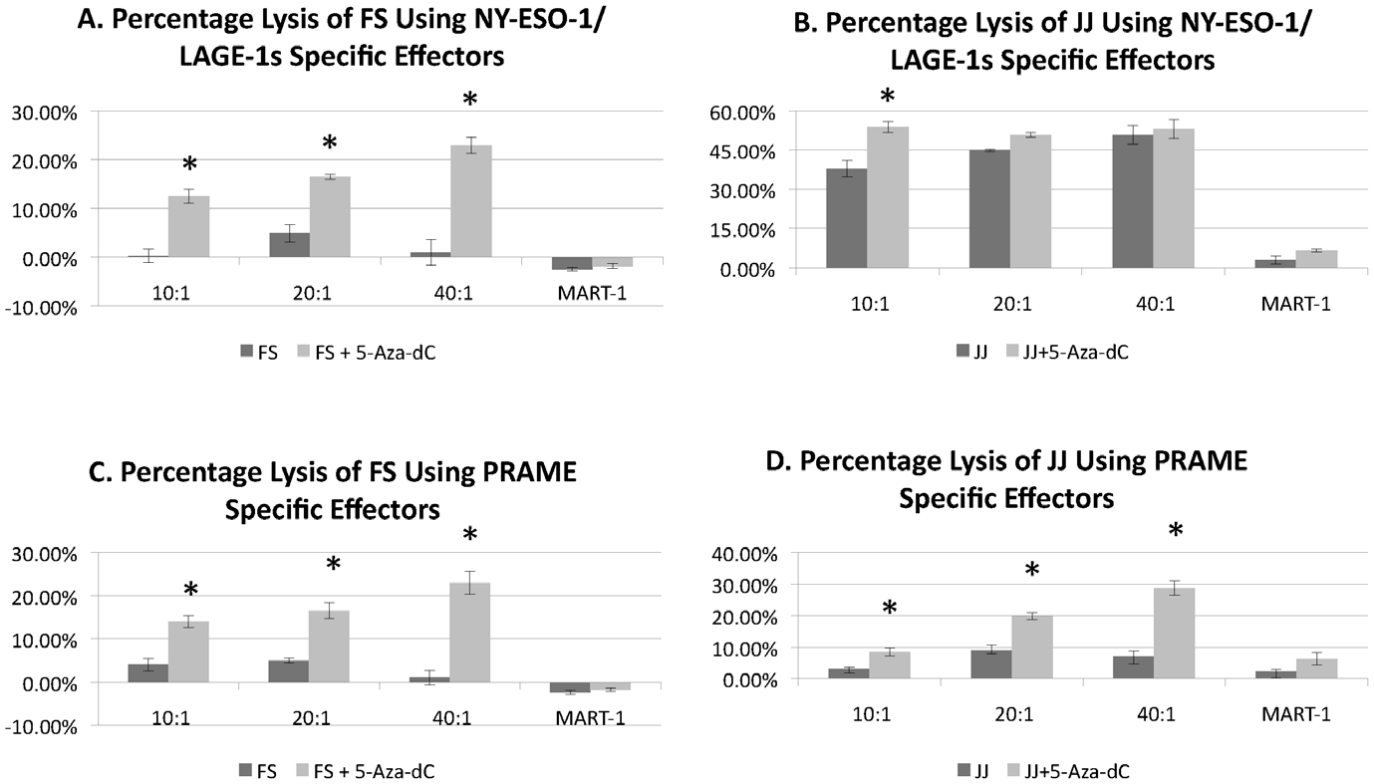
Chromium Release using two A*0201 expressing chondrosarcoma cell lines: FS and JJ. Asterisks indicate statistical significance of p<0.05. Each cell line had significantly increased lysis using antigen specific effectors following treatment with 5-Aza-dC with the exception of JJ in combination with NY-ESO-1/LAGE-1s effectors at high E∶T ratios. JJ is highly sensitive to NY-ESO-1/LAGE-1 specific effectors without 5-Aza-dC treatment and the fact that increased lysis was not seen at high E∶T ratios probably represents a maximum lysis that can be detected for the JJ cell line with these effectors in the given conditions. To control against non-specific cell lysis, targets were tested with a MART-1 specific CTL clone used at an E∶T ratio of 25∶1. Minimal killing was seen testing the control effectors against either treated or untreated targets. No cell line had increased lysis following 5-Aza-dC treatment using the control MART-1 effectors (p>0.1). To be sure the MART-1 specific cells were effective, we also targeted the MART-1 expressing cell line Mel526 during each experiment resulting in over 40% lysis (not shown). Error bars describe variation between multiple values within a single experiment. Experiments were repeated at least three times to ensure reproducibility.

The cell line FS does not express NY-ESO-1 or LAGE-1s. Even at high E∶T ratios such as 100∶1 (not shown) no lysis was seen in this cell line using specific NY-ESO-1/LAGE-1s (SLLMWITQC)-specific CTL. Like FS, the cell line JJ line does not express significant constitutive levels of NY-ESO (barely detectable expression <0.001% of Mel375), however it does express high levels of LAGE-1s and the cell line was readily lysed using the NY-ESO-1/LAGE-1s specific effectors. Even at E∶T ratios as low as 10∶1 over 30% specific lysis (Figure 2A) was seen.

The use of 5-Aza-dC was found to significantly enhance NY-ESO/LAGE-1s mediated killing in the FS cell line compared to cells treated with vehicle alone. Using 5-Aza-dC treated FS targets were lysed over 10% at all E∶T ratios examined and over 20% at the 40∶1 E∶T ratio. The JJ cell line which was lysed even without treatment, was further sensitized to lysis follow 5-Aza-C treatment at low E∶T ratios however at higher E∶T ratios lysis was not increased significantly as lysis of JJ was higher at the higher E∶T ratios with out much change in the lysis of the treated JJ cells. The fact that significantly increased lysis was not seen at high E∶T ratios see probably represents a maximum lysis that can be detected for the JJ cell line with these effectors in the given conditions (Figure 2B).

Using a PRAME specific CTL line, less then 10% of targets were lysed in the untreated JJ and FS cell lines reflecting the low levels of expression seen by qRT-PCR. Following treatment with 5-Aza-dC, increased lysis was seen with both cell lines (Figure 2C and 2D).

In order to confirm that CTL mediated lysis of 5-Aza-dC treated tumor targets were lysed specifically, cold target inhibition was performed (Figure S2). Using both NY-ESO-1 and PRAME specific effectors, lysis of 5-Aza-dC treated JJ and FS was inhibited by peptide pulsed cold targets at the 30∶1 cold to hot target ratio and to lesser extent at the 10∶1 cold to hot target ratio compared with unpulsed controls.

## Discussion

While expression patterns of Cancer Testis Antigens are being increasingly well described in a number of malignancies [29], [30], much work remains to be done in the area of sarcoma and chondrosarcoma in particular [12]. Chondrosarcoma is frequently treatable in the localized setting but for patients with locally advanced/unresectable or metastatic disease there are no proven systemic options. Moreover, high grade and dedifferentiented chondrosarcoma have a high risk of recurrence following resection and this minimal residual disease setting following resection may be the ideal setting for immunotherapies.

Other studies have suggested that immunotherapy could be used to treat chondrosarcoma patients [31], [32]. Indeed lysis of the FS cell line using MAGE-A3 effectors has been documented as has the coexpression of CSAGE along with MAGE-A family antigens [33], [34] though MAGE-A family expression in chondrosarcoma tumors has not been demonstrated at the protein level.

This study is the first to report the targeting of NY-ESO-1/LAGE-1s and/or PRAME in chondrosarcoma. Furthermore the use of 5-Aza-dC to target chondrosarcoma in combiation with antigen-specific immunotherapy is presented for the first time [35], [36].

We report that while a minority of chondrosarcoma patients can be targeted using NY-ESO-1/LAGE-1s or PRAME specific effectors, it may be feasible to broaden the pool of eligible patients by upregulating expression and by enhance targeting of endogenously expressed antigen on tumor cells by pretreatment with 5′Aza-2-Deoxycitadine, (Decitabine), a drug with a well-documented side effect profile (without known autoimmune toxicity) that is considered relatively non-toxic [37].

In murine models, cancer testis antigens are inducible [38] and increased expression has been demonstrated (although antigen presentation has not yet been documented) in human cancer patients in the case of esophageal cancer and lung cancer [20]. These data suggest that adoptive immunotherapy in combination with 5-Aza-dC may be a potential strategy to treat unresectable or metastatic chondrosarcoma patients where no proven systemic therapies exist.

## Supporting Information

Figure S1 **Tetramer and CD8 staining demonstrating tetramer positive populations after CD25 depleted cells are stimulated twice using dendritic cells pulsed with peptide.** Cells were then sorted using tetramer. Cells that were positive for PRAME tetramer were expanded after sorting; cells positive for NY-ESO tetramer were cloned and expanded after sorting.
(PDF)

Figure S2 **5-Aza-dC treated cells from the JJ or FS cells lines were incubated with NY-ESO-1 or PRAME specific effectors along with un-pulsed (control) or peptide pulse T2 lymphocytes.** Killing of the chromium labeled tumor cell lines was completely inhibited at the ratio of 30∶1 cold to hot targets. Killing was also inhibited to a lesser extent at the 10∶1 cold to hot ratio.
(PDF)
