# Supplementary material for: NYESO-1/LAGE-1s and PRAME Are Targets for Antigen Specific T Cells in Chondrosarcoma following Treatment with 5-Aza-2-Deoxycitabine
Source: PLoS One. 2012 Feb 27;7(2):e32165. doi: 10.1371/journal.pone.0032165 (PMC3288075; doi:10.1371/journal.pone.0032165)
Supplement: Figure S1 — Tetramer and CD8 staining demonstrating tetramer positive populations after CD25 depleted cells are stimulated twice using dendritic cells pulsed with peptide. Cells were then sorted using tetramer. Cells that were positive for PRAME tetramer were expanded after sorting; cells positive for NY-ESO tetramer were cloned and expanded after sorting. (PDF) [file pone.0032165.s001.pdf]

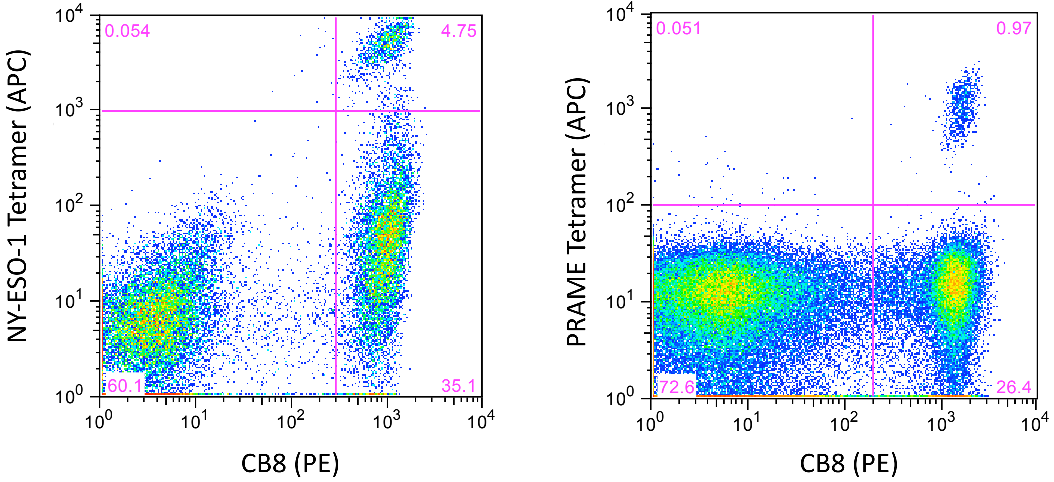

Supplemental Figure: Tetramer and CD8 staining demonstrating tetramer positive populations after CD25 depleted cells are stimulated twice using dendritic cells pulsed with peptide. Cells were then sorted using tetramer. Cells that were positive for PRAME tetramer were expanded after sorting; cells positive for NY-ESO tetramer were cloned and expanded after sorting.
