# Supplementary material for: NYESO-1/LAGE-1s and PRAME Are Targets for Antigen Specific T Cells in Chondrosarcoma following Treatment with 5-Aza-2-Deoxycitabine
Source: PLoS One. 2012 Feb 27;7(2):e32165. doi: 10.1371/journal.pone.0032165 (PMC3288075; doi:10.1371/journal.pone.0032165)
Supplement: Figure S2 — 5-Aza-dC treated cells from the JJ or FS cells lines were incubated with NY-ESO-1 or PRAME specific effectors along with un-pulsed (control) or peptide pulse T2 lymphocytes. Killing of the chromium labeled tumor cell lines was completely inhibited at the ratio of 30∶1 cold to hot targets. Killing was also inhibited to a lesser extent at the 10∶1 cold to hot ratio. (PDF) [file pone.0032165.s002.pdf]

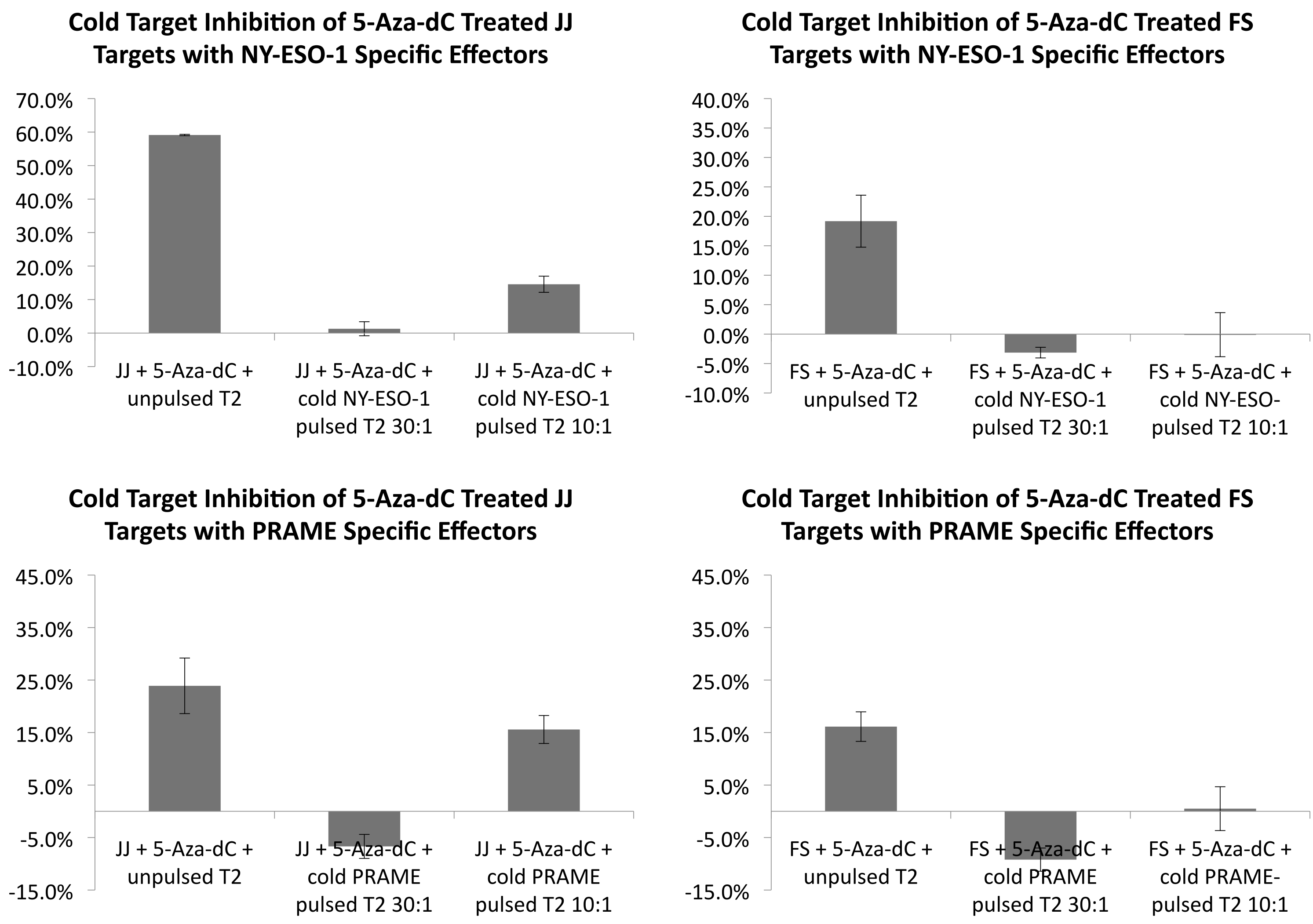

Supplemental Figure 2: 5-Aza-dC treated cells from the JJ or FS cells lines were incubated with NY-ESO-1 or PRAME specific effectors along with un-pulsed (control) or peptide pulse T2 lymphocytes. Killing of the chromium labeled tumor cell lines was completely inhibited at the ratio of 30:1 cold to hot targets. Killing was also inhibited to a lesser extent at the 10:1 cold to hot ratio.
